# Zwitterionic or Not? Fast and Reliable Structure Determination by Combining Crystal Structure Prediction and Solid-State NMR

**DOI:** 10.3390/molecules28041876

**Published:** 2023-02-16

**Authors:** Federica Bravetti, Raffaele E. Russo, Simone Bordignon, Angelo Gallo, Federica Rossi, Carlo Nervi, Roberto Gobetto, Michele R. Chierotti

**Affiliations:** 1 Dipartimento di Chimica e Centro di Eccellenza NIS, Università di Torino, v. P. Giuria 7, 10125 Torino, Italy; 2 Currently at Scuola di Scienze e Tecnologie, Centro di Ricerca ChIP, Università di Camerino, v. Madonna delle Carceri, 62032 Camerino, Italy; 3 Dipartimento di Scienza e Tecnologia del Farmaco, Università di Torino, v. P. Giuria 9, 10125 Torino, Italy

**Keywords:** zwitterionic systems, solid-state NMR, crystal structure prediction, NMR crystallography, N-H distance, structure solution, quinolinic acid, dipicolinic acid, dinicotinic acid

## Abstract

When it comes to crystal structure determination, computational approaches such as Crystal Structure Prediction (CSP) have gained more and more attention since they offer some insight on how atoms and molecules are packed in the solid state, starting from only very basic information without diffraction data. Furthermore, it is well known that the coupling of CSP with solid-state NMR (SSNMR) greatly enhances the performance and the accuracy of the predictive method, leading to the so-called CSP-NMR crystallography (CSP-NMRX). In this paper, we present the successful application of CSP-NMRX to determine the crystal structure of three structural isomers of pyridine dicarboxylic acid, namely quinolinic, dipicolinic and dinicotinic acids, which can be in a zwitterionic form, or not, in the solid state. In a first step, mono- and bidimensional SSNMR spectra, i.e., ^1^H Magic-Angle Spinning (MAS), ^13^C and ^15^N Cross Polarisation Magic-Angle Spinning (CPMAS), ^1^H Double Quantum (DQ) MAS, ^1^H-^13^C HETeronuclear CORrelation (HETCOR), were used to determine the correct molecular structure (i.e., zwitterionic or not) and the local molecular arrangement; at the end, the RMSEs between experimental and computed ^1^H and ^13^C chemical shifts allowed the selection of the correct predicted structure for each system. Interestingly, while quinolinic and dipicolinic acids are zwitterionic and non-zwitterionic, respectively, in the solid state, dinicotinic acid exhibits in its crystal structure a “zwitterionic-non-zwitterionic continuum state” in which the proton is shared between the carboxylic moiety and the pyridinic nitrogen. Very refined SSNMR experiments were carried out, i.e., ^14^N-^1^H Phase-Modulated (PM) pulse and Rotational-Echo Saturation-Pulse Double-Resonance (RESPDOR), to provide an accurate N–H distance value confirming the hybrid nature of the molecule. The CSP-NMRX method showed a remarkable match between the selected structures and the experimental ones. The correct molecular input provided by SSNMR reduced the number of CSP calculations to be performed, leading to different predicted structures, while RMSEs provided an independent parameter with respect to the computed energy for the selection of the best candidate.

## 1. Introduction

In crystal engineering, single-crystal X-ray diffraction (SCXRD) is definitely the most used technique for the determination of crystal structures, even if structure determination from powder data, via direct-space methods or more sophisticated methods, like fit to the pair distribution function (PDF) [1], have become more and more reliable. In cases where these techniques may be unfeasible, predicting how molecules will pack in space becomes crucial. Despite many efforts, this often proves to be quite a hard task and represents a still open challenge. In this context, computational methods, such as Crystal Structure Prediction (CSP), have taken hold more and more in the last decades [2,3,4]. Originally developed for screening the polymorph landscape [5,6,7,8], CSP has proved to be a very useful tool for crystal structure determination as well, as confirmed also by the results of six blind tests [9] promoted by the Cambridge Crystallographic Data Centre (CCDC). However, CSP still exhibits some limitations, such as the fact that it does not consider the effects due to temperature [10] (calculations are usually performed at 0 K) or that all predicted structures are generally optimised by ab initio quantum mechanical methods, requiring considerable computational resources which increase with the system size. Moreover, theoretically, numerous CSP calculations are required if information such as the number of independent molecules in the unit cell (*Z*′), tautomeric or zwitterionic character or conformations are missing. Thus, a valid option is to couple CSP to experimental techniques, e.g., exploiting structural information about the unit cell or local structure derived from powder X-ray diffraction (PXRD) or solid-state nuclear magnetic resonance (SSNMR).

SSNMR measurements on crystalline samples have long proved to be crucial for structural characterisation and definition of hydrogen bond patterns, an approach known as NMR crystallography (NMRX) [11,12,13]. NMRX is indeed applied in combination with diffraction methods, especially with powder diffraction, since it does not require long-range order and can be a valuable resource for studying atom–atom proximities and distances, disorder effects, heterogeneous phases, polymorphism, etc. Either alone or combined with XRD and/or calculations, it has been used for solving the structure of organic molecules [14,15,16,17,18]. Furthermore, the high sensitivity of SSNMR to the ^1^H nucleus offers unambiguous information about the protonation state of the crystal forms, allowing for the discrimination between salts and co-crystals [19], or different tautomeric forms [20].

Hence, CSP finds its perfect partner in SSNMR, leading to the so-called CSP-NMR crystallography (CSP-NMRX) approach, in which SSNMR experiments can provide information such as the number of independent molecules in the unit cell (*Z*′), conformation [17,21] and neutral or ionic character [22,23], exploitable as constraints that reduce the search space [24] and improve the reliability of the predicted structures. Furthermore, SSNMR can play a key role also for the selection of the correct structure among all predicted ones by comparing experimental and computed chemical shifts [25,26]. In 2019, Hofstetter et al. presented the crystal structure prediction of ampicillin guided by SSNMR information [27]. They used NMRX to find the molecular conformation of ampicillin in the solid state, which was different from the lowest energy conformation obtained by common gas-phase optimisations. We successfully applied this combined method on mebendazole [28], an antihelmintic drug, which presents five possible tautomers and three desmotropes, i.e., crystal phases in which different tautomers are isolated [29].

**Scheme 1:**
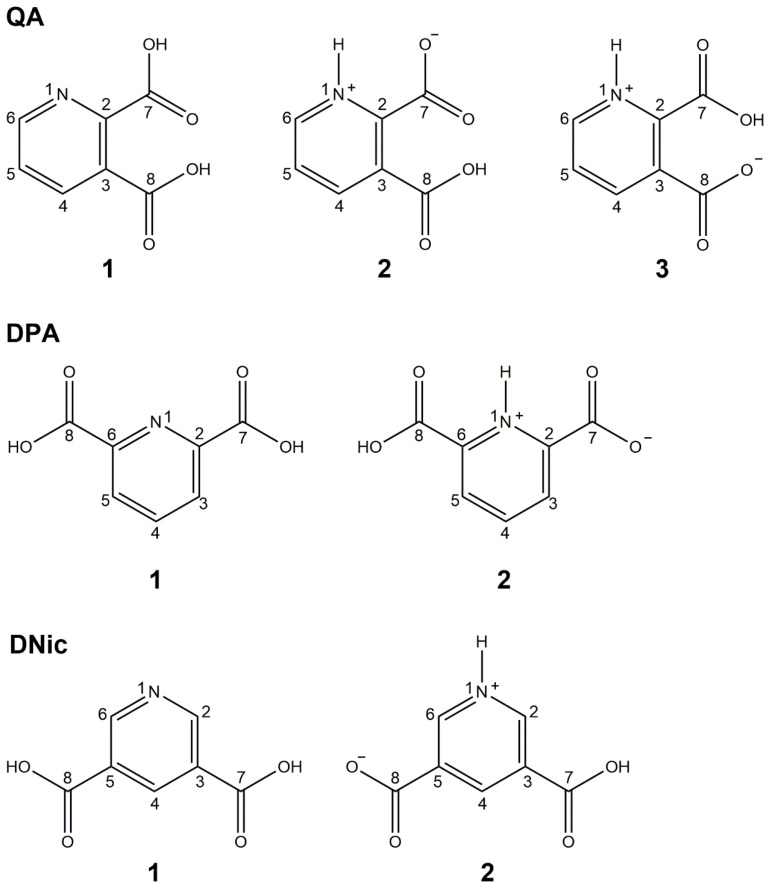
Molecular structures of possible zwitterionic and non-zwitterionic quinolinic acid (QA), dinicotinic acid (DNic) and dipicolinic acid (DPA). For QA, also the two possible isomers of the zwitterionic form are reported.

Here, we extend the applicability of this approach to potential zwitterionic systems, namely quinolinic (QA, pyridine-2,3-dicarboxylic acid), dinicotinic (DNic, pyridine-3,5-dicarboxylic acid) and dipicolinic (DPA, pyridine-2,6-dicarboxylic acid) acids. They were selected as model samples to test the efficiency and accuracy of the CSP-NMRX method since they are three structural isomers of pyridine dicarboxylic acid with molecular formula C_7_H_5_NO_4_ (Scheme 1), widely used in the construction of coordination polymers, and because their metal coordination abilities allow for different types of architectures [30,31]. Furthermore, such systems have the peculiarity to be potentially zwitterionic in the solid state (Scheme 1) and each crystallises with a different protonation state. In particular, for the zwitterionic form of QA, two different isomers are possible, with the carboxylate group either on C7 or on C8 (structures 2 and 3 in Scheme 1, respectively).

**Figure 1:**
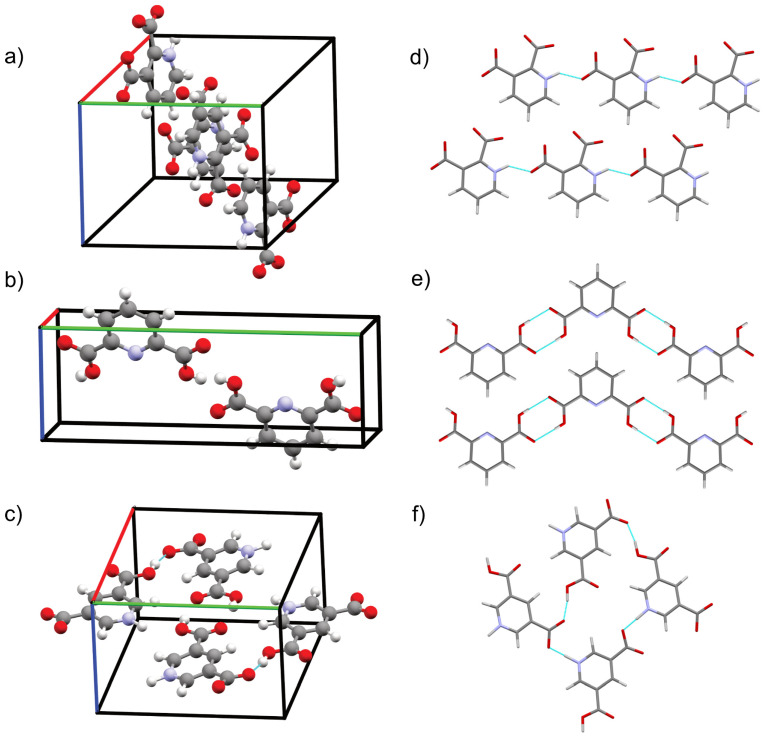
Unit cells of the experimental crystal structures of (**a**) QA, (**b**) DPA and (**c**) DNic. View direction [100]; hydrogen bond patterns in the crystal structures of (**d**) QA, (**e**) DPA and (**f**) DNic (15 K). Colour code in all drawings: C = grey, O = red, N = blue, H = white, hydrogen bonds = turquoise.

The crystal structures of these systems, solved by single-crystal X-ray diffraction (SCXRD) at different temperatures, were already reported in literature (Figure 1a–c), while no polymorphs are known.

QA (Figure 1a, space group *P*2_1_/*c* (14), *Z*′ = 1, CSD refcode QUICNA10) crystallises in the zwitterionic form [32], with the transfer of the carboxylic hydrogen to the pyridinic nitrogen. This was confirmed by performing SCXRD analyses at both ambient and low temperatures (100, 80 and 35 K) [33]. In the crystal structure of QA, the molecules are arranged in layers (Figure 1d); each molecule on the same plane is connected to the others by N^+^-H∙∙∙O^−^ intermolecular hydrogen bonds between adjacent carboxylic groups (O–N distance = 2.725 Å).

DPA (Figure 1b, space group *P*2_1_/*m* (11), *Z*′ = 1, CSD refcode AFEBUI) shows instead a non-zwitterionic character in the solid state [34]. In the crystal structure of DPA, the molecules are arranged with a ribbon-like shape (Figure 1e). The molecules are connected in infinite chains by homosynthonic hydrogen bonds, $R_{2}^{2}\left(8\right)$, between adjacent carboxylic groups (O···O distance = 2.625 Å).

Regarding DNic (Figure 1c, space group *P*2_1_/*c* (14), *Z*′ = 1, CSD refcode DINICA12), neutron diffraction (ND) experiments at room temperature (RT) revealed that DNic seems to be in a “continuum” state, with the proton shared between the carboxyl and the nitrogen [35]. At a very low temperature (15 K, CSD refcode DINICA11) [36] the proton moves towards the pyridinic nitrogen, resulting in a more pronounced, but still not complete, zwitterionic form. In the crystal structures of DNic, the molecules lie in parallel planes. On the same plane (Figure 1f), they are connected by two kinds of hydrogen bonds: one between two carboxylic groups (O···O distance = 2.594 Å at RT) and one between a carboxylic group and the pyridinic nitrogen, N∙∙∙H∙∙∙O (O···N distance = 2.515 Å at RT), forming an $R_{4}^{4}\left(24\right)$ hydrogen-bonded ring.

The analysis of these systems by means of SSNMR proved crucial to determine the protonation state, which was used as a constraint in the CSP calculations and led to the successful prediction of the crystal structures of QA, DPA and DNic.

This paper addresses the following key points and novelties:

- The development of a method for rapid determination of crystal structures of possible zwitterionic systems, with the extent of applicability of this approach being nonetheless much broader than that of zwitterionic systems, since it covers, in general, organic, organometallic and inorganic compounds for which the crystal structure is not available and which have uncertain proton positions;
- The unambiguous elucidation of the zwitterionic character of the three structural isomers of pyridine dicarboxylic acid;
- The evaluation of the “zwitterionic–non-zwitterionic continuum state” in dinicotinic acid with very accurate NMR measurements of the N-H distance. Also in this case, the method can be easily applied to other known situations, such as, for instance, the “salt-cocrystal continuum”.

## 2. Results and Discussion

### 2.1. SSNMR Analysis: Zwitterionic Character

QA, DPA and DNic were firstly analysed by SSNMR, to collect information about the content of the unit cell, such as the value of *Z*′ and the zwitterionic character (i.e., protonation state of carboxylic and pyridinic groups). This NMR analysis has contributed enormously to identify the correct structure to submit for the CSP calculations. Mono- (^1^H MAS, ^13^C and ^15^N CPMAS) and bidimensional (^1^H-^13^C short- and long-range HETCOR, ^1^H DQ MAS) experiments were conducted at room temperature for all samples (see Table S1 in the Supplementary Materials for all experimental parameters). For convenience, also the comparisons among ^1^H, ^13^C and ^15^N spectra are reported separately in the Supplementary Materials, Figures S1–S3, respectively. The ^1^H, ^13^C and ^15^N SSNMR chemical shifts are well known to be quite sensitive to protonic transfer, in particular for the heteronuclei (^13^C and ^15^N) in carboxylic and pyridinic moieties involved in the resulting hydrogen bonds [37]. The presence of hydrogen bonds in the crystals was assessed by ^1^H MAS spectra; indeed, in general, all hydrogen-bonded ^1^H chemical shifts appear to be higher for shorter distances between heavy atoms (i.e., stronger hydrogen bonds) and vice versa, in agreement with the literature [37,38,39]. All chemical shifts (^1^H, ^13^C and ^15^N) are reported in detail in the Supplementary Materials (Tables S2–S4) while the comparisons between chemical shifts and distances between heavy atoms are reported in Table 1.

#### 2.1.1. Quinolinic Acid

**Figure 2:**
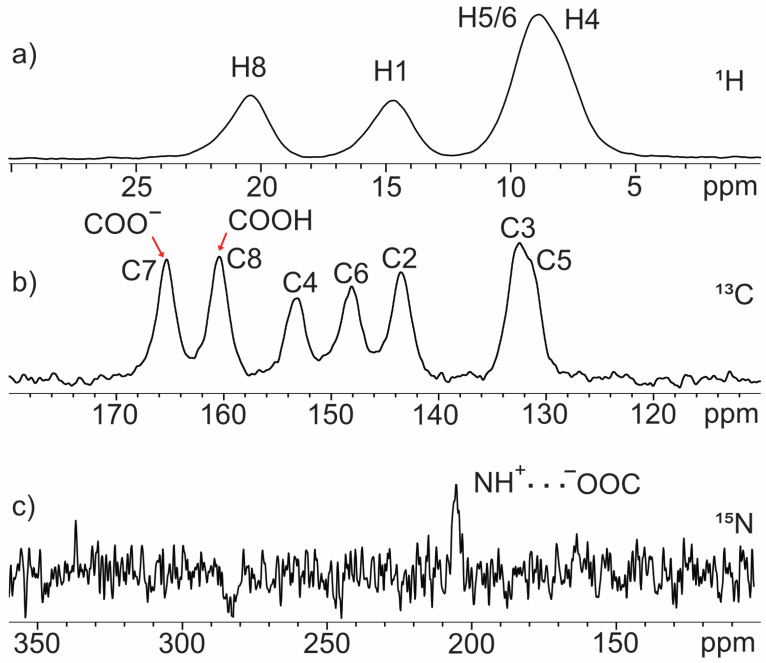
^1^H (600.17 MHz) MAS (**a**), ^13^C (100.63 MHz) (**b**) and ^15^N (40.56 MHz) (**c**) CPMAS spectra of QA acquired at room temperature at a spinning speed of 50 kHz for ^1^H, 12 kHz for ^13^C and 9 kHz for ^15^N. Signals assignments refer to Scheme 1.

The ^1^H MAS, ^13^C and ^15^N CPMAS SSNMR spectra (Figure 2) of QA clearly report the presence of only one independent molecule in the unit cell (*Z*′ = 1), since they are characterised by only one set of signals (i.e., seven peaks out of seven expected in the ^13^C spectrum and 1 peak out of 1 expected for the ^15^N spectrum). Specifically, in the ^13^C CPMAS spectrum, the resonances at 160.4 and 165.3 ppm are ascribable to carboxylic groups and, in particular, the resonance at lower ppm values is typical of a free neutral COOH group, while that at higher frequencies accounts for a deprotonated (COO^−^) group, or a neutral group possibly involved in a hydrogen bond.

In the ^1^H MAS spectrum, the hydrogen-bond region (above 10 ppm) is characterised by one signal at 14.7 ppm (H1) and another one at 20.4 ppm (H8); the former is consistent with a pyridinium proton involved in a hydrogen bond of intermediate strength, the latter with the presence of a COOH group involved in very strong hydrogen bonds, usually an intramolecular interaction [37,38,39]; this agrees with both the intramolecular hydrogen bond between carboxylic groups present in the experimental crystal structure of the system and the short distance between heavy atoms (see Table 1).

To further validate the presence of a hydrogen bond for the pyridinic moiety and investigate its protonation state, we also acquired a ^15^N CPMAS spectrum of QA. It clearly shows a signal at 205.3 ppm, characteristic of a protonated pyridinic nitrogen, i.e., about 100 ppm lower in frequency with respect to that of the free pyridinic nitrogen (around 300–320 ppm, see DPA below) [37].

**Figure 3:**
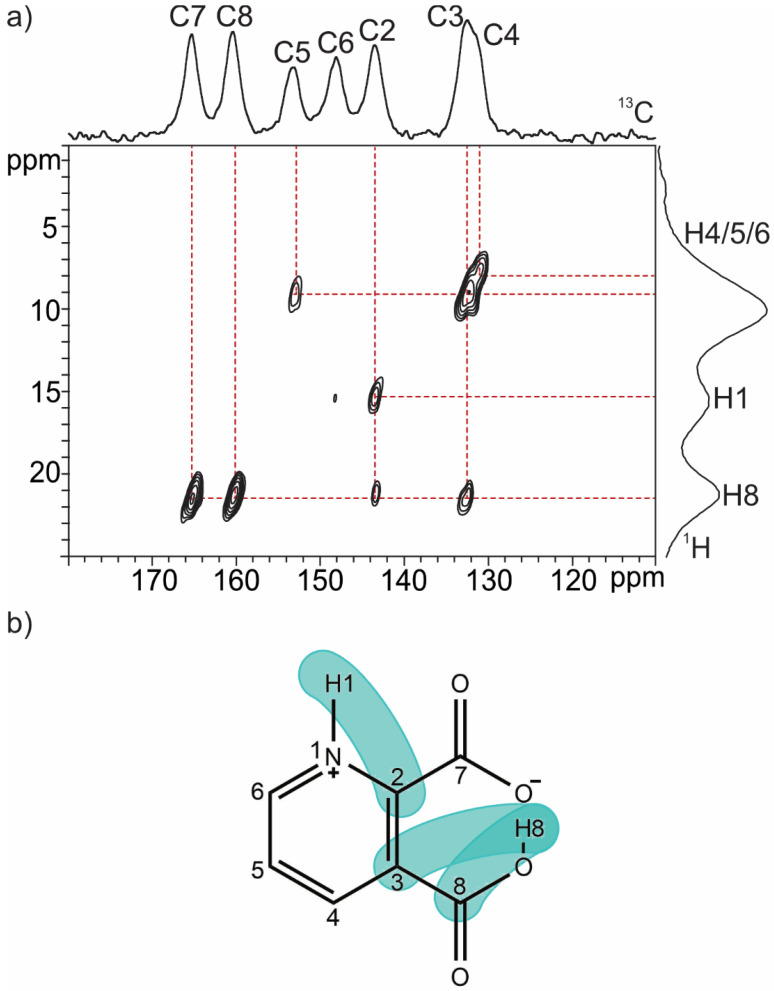
(**a**) 2D long-range ^1^H-^13^C FSLG HETCOR SSNMR spectrum of QA. (**b**) Scheme of the main spatial proximities as found in the long-range ^1^H-^13^C HETCOR spectra. Only the main correlations of the two protons involved in hydrogen bonds, H8 and H1, are highlighted in jade green.

This aggregated information, clearly encoded in the SSNMR spectra, shows that QA is in a zwitterionic form in its crystal structure. However, the position of the carboxylate group on the ring was still unclear, since it could be either in the ortho (C2) or meta (C3) positions relative to the pyridinic N. Thus, 2D short- (Figure S4 in the Supplementary Materials) and long-range (Figure 3a) ^1^H-^13^C FSLG HETCOR spectra were performed to univocally define the assignment of ^13^C signals.

Magnetisation transfer is observed from H8 to C2, C3, C7 and C8, and from H1 to C2 (Figure 3). In particular, the former suggests the formation of an intramolecular hydrogen bond between the COOH (and its hydrogen H8) and the carboxylate group. Indeed, for an intermolecular bond, the distance would have been greater than 3.0 Å, which is incompatible with the presence of a correlation. This hypothesis is strongly supported by the ^1^H MAS analysis (Figure 2a) reported above. Furthermore, the higher intensity of the H8/C3 correlation with respect to the H8/C2 one suggests that the carboxylic group is in the meta (C3–C8) rather than in the ortho (C2–C7) position. Although qualitative, this evidence is supported by the use of the off-resonance CP (LG-CP), which avoids spin diffusion and guarantees that the polarisation transfer occurs by heteronuclear dipolar interaction only.

All these NMR data are in perfect agreement with the SCXRD structure, which shows QA in the zwitterionic form, with the carboxylate group in the ortho position.

#### 2.1.2. Dipicolinic Acid

**Figure 4:**
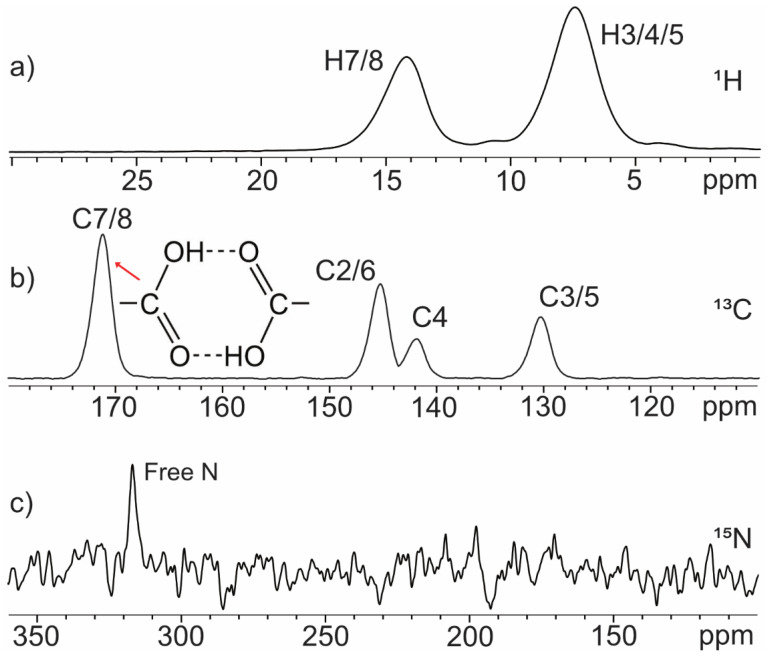
^1^H (600.17 MHz) MAS, ^13^C (100.63 MHz) CPMAS (**a**), (**b**) and ^15^N (40.56 MHz) CPMAS (**c**) spectra of DPA acquired at room temperature at a spinning speed of 68 kHz for ^1^H, 12 kHz for ^13^C and 9 kHz for ^15^N, respectively. Signals assignments refer to Scheme 1.

In the DPA ^13^C CPMAS spectrum (Figure 4b), only four signals are present over seven carbon atoms, implying a high level of symmetry of the molecule in the unit cell. Indeed, the resonance at 171.2 ppm, which integrates for two, is ascribable to two carboxylic groups. Therefore, we can assess the presence of one independent molecule in the unit cell (*Z*′ = 1). Furthermore, the carboxylic groups are probably involved in an $R_{2}^{2}\left(8\right)$ homosynthonic hydrogen-bond pattern.

**Figure 5:**
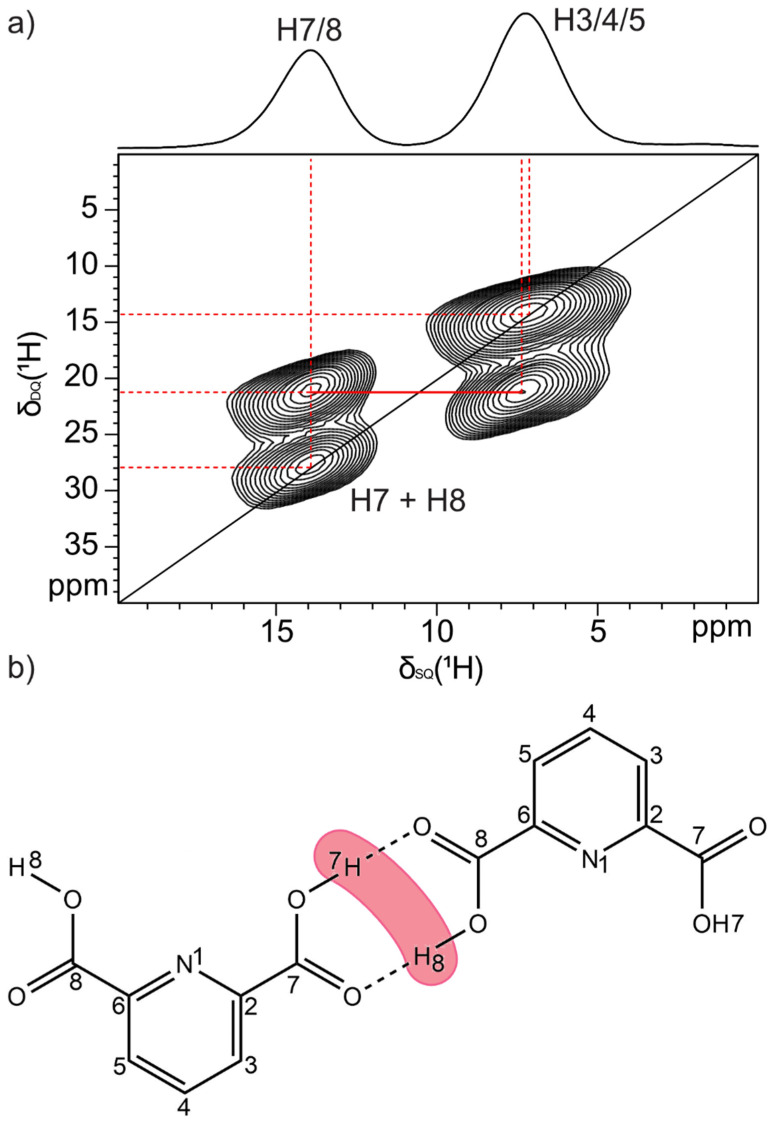
(**a**) ^1^H (600.17 MHz) DQ MAS spectrum of DPA recorded at 68 kHz. (**b**) Scheme of the main spatial proximities related to the carboxylic hydrogen atoms discussed in the text.

The ^1^H MAS spectrum (Figure 4a) was once more instrumental to ascertain the presence of intermolecular hydrogen bonds. Indeed, the ^1^H signal at 14.2 ppm (H7/H8), integrating for 2 protons, and the autocorrelation cross-peak at δ_DQ_ = 14.2 + 14.2 = 28.4 ppm (^1^H) in the ^1^H DQ MAS spectrum (Figure 5), are consistent with the presence of two COOH groups involved in the $R_{2}^{2}\left(8\right)$ homosynthonic hydrogen-bond pattern of intermediate strength. On the contrary, the absence of hydrogen bonds involving the pyridinic group is confirmed by the ^15^N spectrum (Figure 4c), showing only a signal at 316.0 ppm, a chemical shift characteristic of a free pyridinic nitrogen [37].

**Figure 6:**
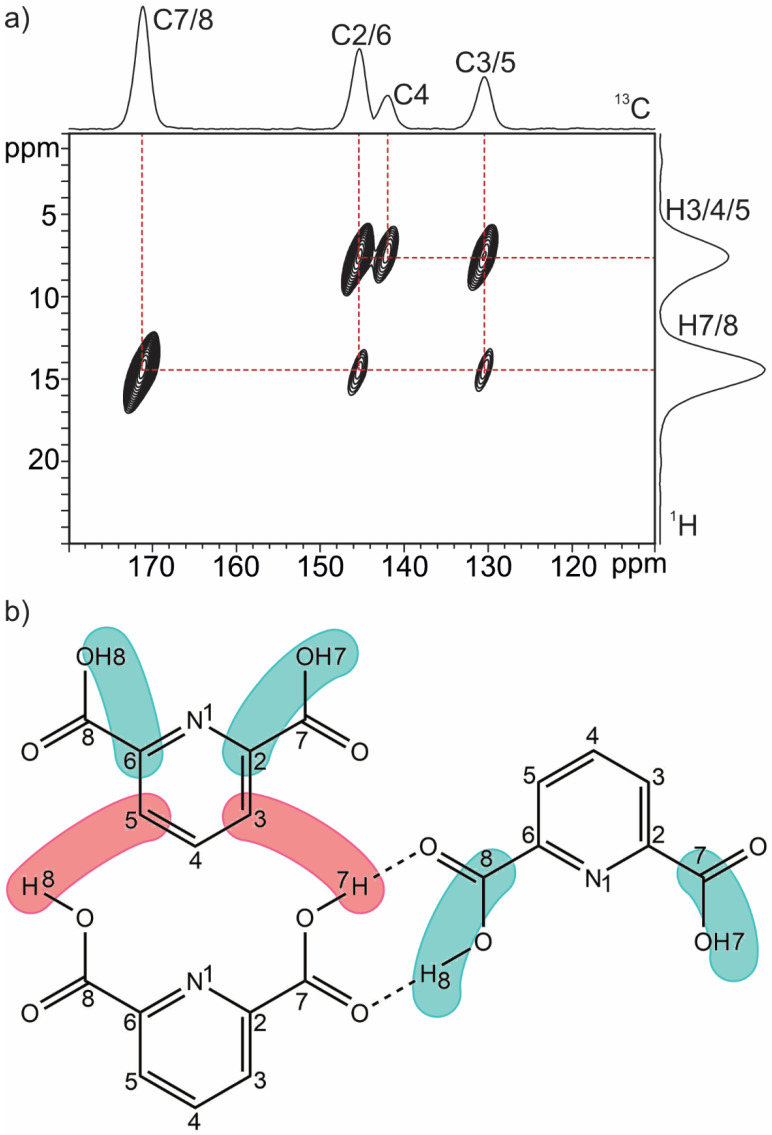
(**a**) 2D long-range ^1^H-^13^C FSLG HETCOR SSNMR spectrum of DPA. Only the most relevant correlations are highlighted. (**b**) Scheme of the main intra- (jade green) and intermolecular (red) spatial proximities, as found in the ^1^H-^13^C long-range HETCOR spectra.

2D short- (Figure S5 in the Supplementary Materials) and long-range (Figure 6a) ^1^H-^13^C HETCOR spectra corroborated the conclusions drawn from 1D measurements, ultimately establishing the neutral character of the system.

Furthermore, some spatial proximities highlighted in Figure 6b give some important information about the crystal packing. In detail, the detected H8/C5 and H3/C7 correlations can only refer to intermolecular proximities, not compatible with the long interatomic intramolecular distances between the involved heavy nuclei.

#### 2.1.3. Dinicotinic Acid

**Figure 7:**
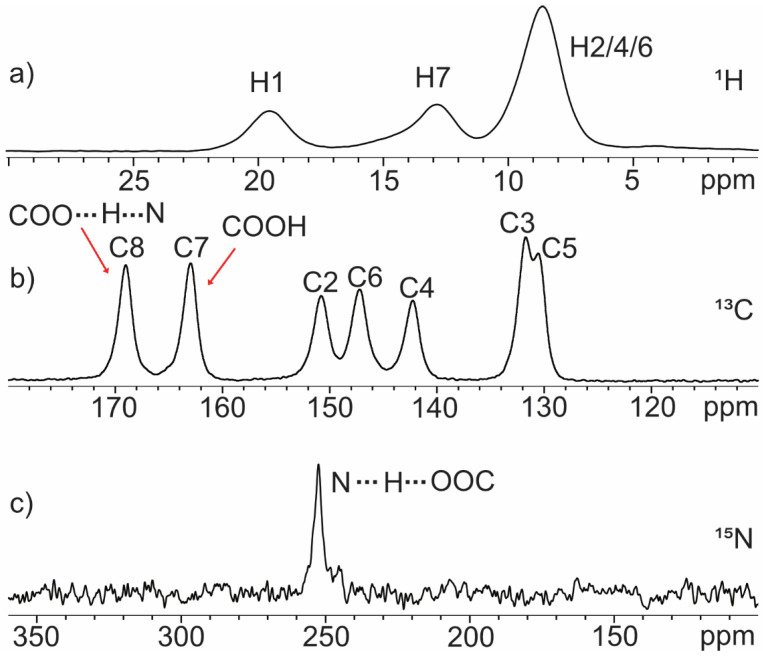
^1^H (600.17 MHz) MAS (**a**), ^13^C (100.63 MHz) CPMAS (**b**) and ^15^N (40.56 MHz) CPMAS (**c**) spectra of DNic acquired at room temperature at a spinning speed of 60 kHz for ^1^H, 12 kHz for ^13^C and 9 kHz for ^15^N, respectively. Signals assignments refer to Scheme 1.

As in the previously discussed cases, the ^13^C CPMAS spectrum of DNic (Figure 7b) indicates the presence of one independent molecule in the unit cell (*Z*′ = 1), since the number of expected peaks (7) matches exactly the number of carbon nuclei in the molecule (7). Focusing on the carboxylic region, two distinct resonances are observed at 162.9 and 169.0 ppm. The peak at lower ppm values is related to a carboxyl group (COOH), while the latter can be assigned to either a carboxylate or a carboxyl group involved in a strong hydrogen bond or even an intermediate situation between a carboxylic and a carboxylate group, i.e., a continuum in which the proton is shared between the oxygen and the nitrogen atoms.

To validate the information obtained from the ^13^C spectrum and to explore in more detail the nature of this hydrogen bond, we also performed and analysed 1D ^1^H MAS and 2D ^1^H DQ MAS spectra (Figure 7a and Figure S6 in the Supplementary Materials). As for the other two investigated systems, the hydrogen-bond region exhibits one signal at 12.8 ppm (H7), consistent with a COOH proton involved in a hydrogen bond; the high-frequency resonance at 19.6 ppm, associated to H1, agrees with the presence of a strong hydrogen bond (see Table 1).

Additionally, we looked at the ^15^N CPMAS spectrum to precisely determine the protonation state of the compound in its crystal structure (i.e., zwitterionic, neutral or continuum). The ^15^N spectrum displays a resonance at 252.9 ppm, which is an intermediate value between those of QA (205.3 ppm) and DPA (316.0 ppm), and usually is associated to a neutral pyridinic nitrogen involved in a hydrogen bond [37]. In this specific instance, no definitive hypothesis could thus be made around the protonation state of DNic based only on the 1D spectra, which led to the acquisition of the 2D ^1^H-^13^C correlation experiments and N–H distance measurements.

**Figure 8:**
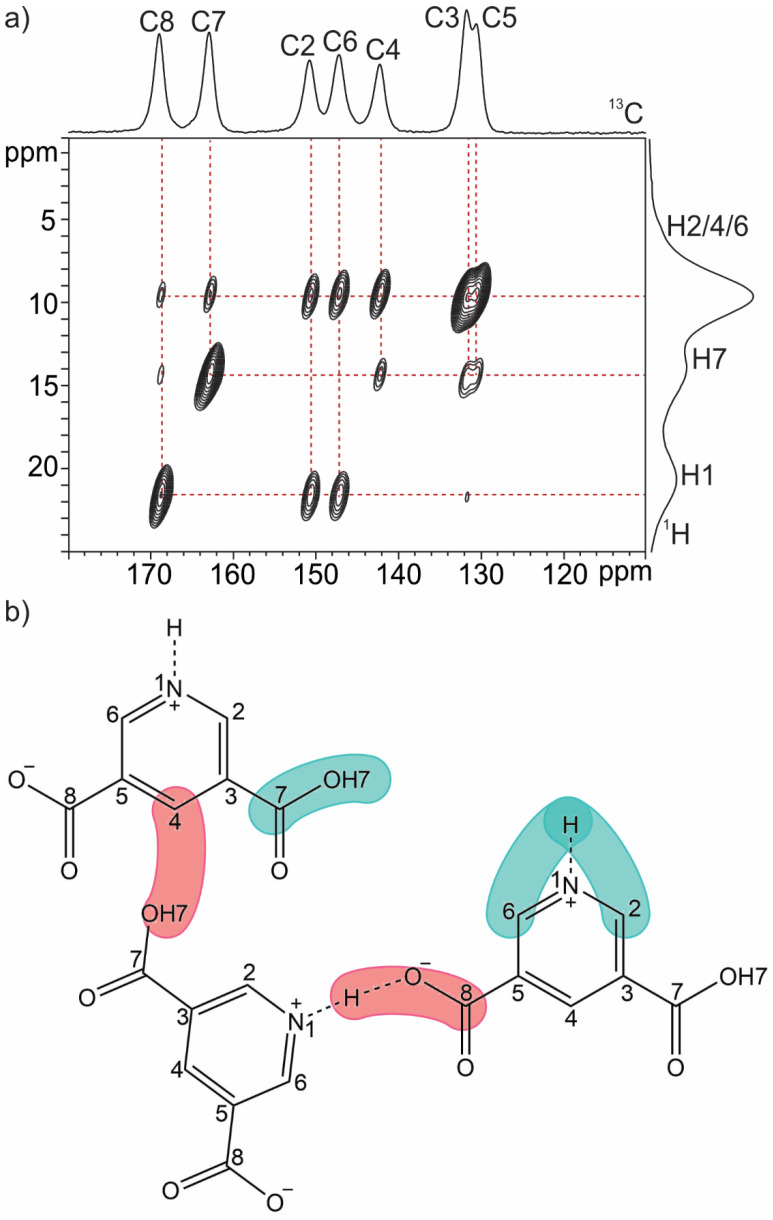
(**a**) 2D long-range ^1^H-^13^C FSLG HETCOR SSNMR spectrum of DNic. Only the most relevant correlations are highlighted. (**b**) Scheme of the main intra- (jade-green) and intermolecular (red) spatial proximities as found in the ^1^H-^13^C long-range HETCOR spectra.

The 2D short-range (Figure S7 in the Supplementary Materials) and long-range (Figure 8a) ^1^H-^13^C FSLG HETCOR SSNMR spectra of DNic clearly show that the H7/C3-C4-C5 and H7/C8 correlations are associated to intermolecular proximities, since the interatomic distances within the molecule would be greater than 4 Å.

Thus, these proximities provide an idea of the spatial arrangement of the molecules in the unit cell. The intensities of the correlations between H1 and C2 and C6 suggest that H1 may be very close to the pyridinic nitrogen, which could even be protonated, in agreement with the 1D ^13^C data. Indeed, the strong H1/C8 correlation is fully consistent with the formation of a hydrogen bond between the carboxylic group (C8) and N1.

The presence of the H7/C3 correlation, most likely due to an intramolecular proximity, and the absence of a similar correlation between H8 and C5 led to the conclusion that the molecule in the unit cell might be zwitterionic, with C7 corresponding to a neutral carboxylic moiety. As for C8, from all the SSNMR spectra shown above, it is still not clear whether the molecule is to be considered properly zwitterionic or whether the proton is shared. Indeed, this last hypothesis would agree with the ND crystal structure at room temperature, in which a neutral-zwitterionic continuum is observed (where “neutral” stands for a species without charge separation).

To clearly define the position of the proton along the axis of this hydrogen bond and resolve this doubt, we exploited the NMR technique to its full potential and a ^14^N-^1^H PM-S-RESPDOR experiment was performed to precisely measure the N–H distance. Indeed, it has been proved that this sequence is able to extract reliable N–H distances at natural abundance [40,41,42], having been also successfully tested on real samples [22]. Details of the experimental procedure for the extraction of the N–H distance are reported in the Supplementary Materials.

**Figure 9:**
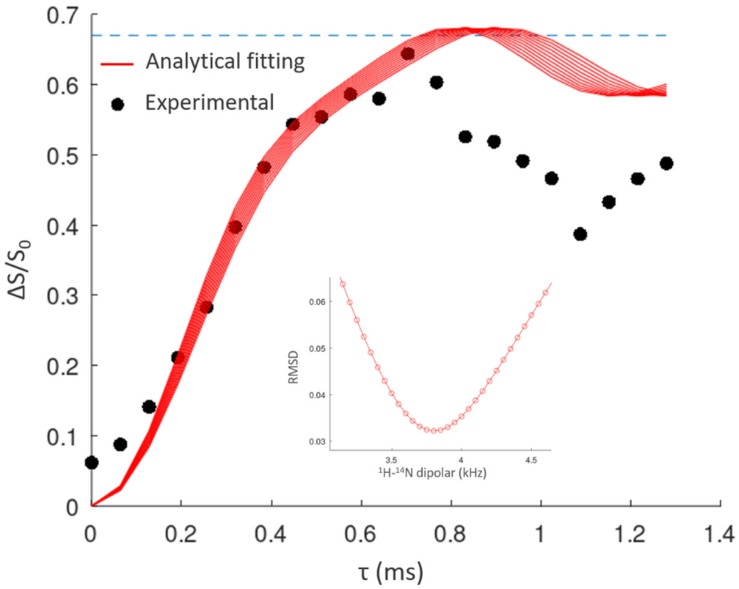
Experimental ^14^N–^1^H Δ*S*/*S*_0_ fraction curves (black dots) achieved by the PM-S-RESPDOR experiment on DNic at the ^1^H chemical shift of 19.6 ppm and analytical fitting curves (red solid lines). The inset shows the best fitting ^1^H–^14^N dipolar coupling constant on the basis of root-mean-square deviation analysis.

The N–H distance is extracted by matching the experimental and analytical fitting of the Δ*S*/*S*_0_ fraction curve of the ^1^H signal of H1 at 19.6 ppm, as assigned from the 2D long-range ^1^H-^13^C FSLG HETCOR SSNMR spectrum. The extracted value was obtained by considering the mixing time (τ) value up to 0.8 ms. The experimental and simulated Δ*S*/*S*_0_ fraction curves are represented in Figure 9 as black dots and solid red lines, respectively. The best fittings are obtained with an N–H distance of 1.31 Å which is highly comparable to the value measured in the ND crystal structure of 1.308 Å [36]. This measurement clearly indicates that the proton is shared between the pyridinic nitrogen and the oxygen of the C8 carboxylic group, to form a neutral-zwitterionic continuum. This case proves SSNMR to be an excellent and elegant tool for precisely determining the position of protons also in ambiguous systems such as DNic.

**Figure 10:**
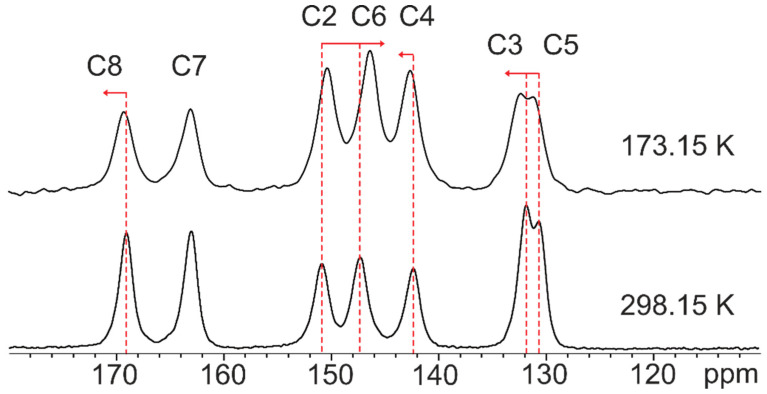
^13^C CPMAS spectra of DNic acquired at room (bottom) and low (top) temperature.

A further piece of information that upholds the shared-proton nature of this hydrogen bond comes from a ^13^C CPMAS spectrum of DNic recorded at 173.15 K (Figure 10). Indeed, previous studies illustrated how in carboxylic acid/pyridine adducts the shared proton moves closer to the nitrogen site with decreasing the temperature [43,44]. The complete list of chemical shifts is reported in Table S4 in the Supplementary Materials.

The low-temperature spectrum shows some small but reliable differences concerning the aromatic peaks of C2, C4 and C6. A minor shift of the C8 one to higher frequencies (Δδ = 0.3 ppm) is also observed. The direction of the shift is consistent with the low-temperature (15 K) ND structure, in which the hydrogen moves towards the nitrogen atom (N–H distance: 1.213 Å), i.e., towards a zwitterionic state. The small entity of this shift (0.3 ppm) appears sound in three different aspects: (i) the SSNMR spectrum was acquired at 173 K and not at 15 K; (ii) at 15 K the ND still shows an intermediate and not a complete zwitterionic character, i.e., N–H distance = 1.213 Å rather than the usual 1.128 Å expected for N^+^-H as extrapolated from a CSD survey (CSD version 5.40, updated in September 2019 on the N–H and N–D distances of pyridine–carboxylic acid interactions in ND structures); (iii) at 173 K, the hydrogen may still have some dynamic features and NMR could be observing an averaged situation.

### 2.2. CSP

Thanks to the information obtained by SSNMR, we were able to define the correct input for the CSP calculation of QA and DPA (i.e., *Z*′ = 1 for both and zwitterionic and non-zwitterionic character in the solid state, respectively). In the case of QA we started from the zwitterionic structure with the carboxylate group in C7 and the carboxylic group in C8. Concerning DNic, since from SSNMR data the proton seemed to be shared between the carboxylic group and the pyridinic nitrogen, but with a continuous shift towards the nitrogen with decreasing the temperature, we used the zwitterionic form as molecular input for the CSP calculation.

The individuals (IDs) generated by the CSP were clustered based on the degree of overlap using the Mercury utility Crystal Packing Similarity. The best IDs with *Z*′ = 1 were identified in a range of 10 kJ/mol, i.e., the energy range in which polymorphs can be found [45], and subsequently re-optimised at DFT level with QE, also performing the chemical shifts calculation.

#### 2.2.1. CSP of QA

The CSP calculation produced 1064 IDs. The best IDs, in the range of 10 kJ/mol, are reported in Table 2. After the DFT optimisation step, only two IDs (315 and 630) were included in the prefixed energy range of 10 kJ/mol. For these IDs, SSNMR chemical shifts were calculated and the ^13^C and ^1^H RMSEs between computed and experimental chemical shifts were evaluated, as reported in Table 2.

ID 315 seemed to be slightly thermodynamically favoured, but the small energy difference and its higher ^13^C and ^1^H RMSE values indicate that ID 630 should be preferred as a candidate for the final structure. A comparison of cell parameters of the experimental and the predicted crystal structure is reported in Table 3. Experimental and computed (ID 630) cell volume, axes lengths, and angles show an overall remarkable agreement.

**Figure 11:**
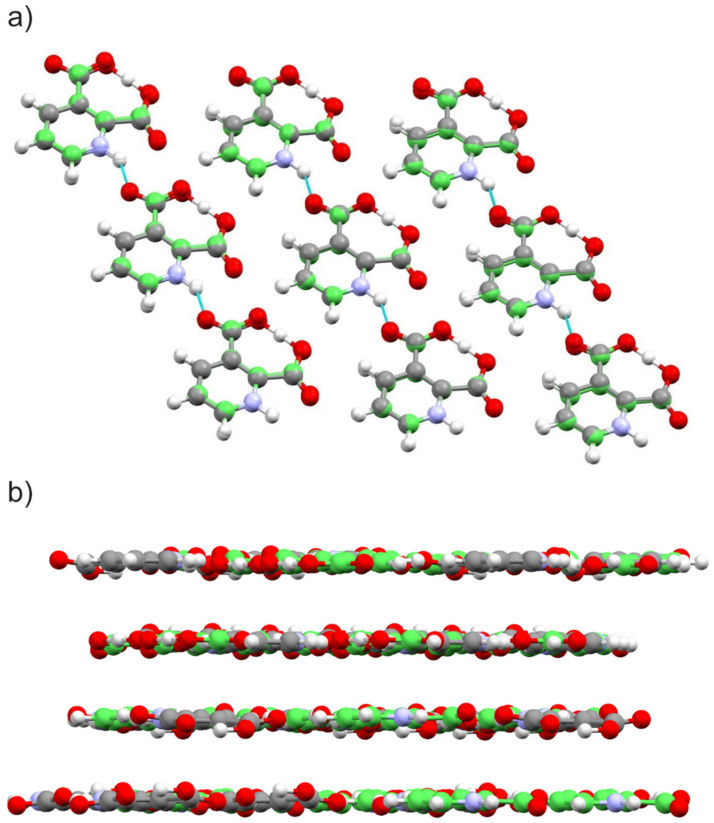
Overlay of the experimental crystal structure of QA (standard colours: C = grey, O = red, N = blue, H = white, hydrogen bonds = turquoise) and the best predicted individual (ID 630, in green): (**a**) hydrogen-bond pattern and (**b**) molecular layers.

The overlay of ID 630, the one selected according to the SSNMR RMSEs, and the experimental structure of QA, shown in Figure 11, points out the almost perfect correspondence between hydrogen-bond patterns and molecular arrangements. A remarkably low RMSE (0.132 Å) of the overlay of these two molecular packing is observed.

#### 2.2.2. CSP of DPA

DPA crystallises in the neutral form with *Z*′ = 1, as revealed by SSNMR; thus, this information was used as CSP input. A total of 1089 IDs were produced by USPEX, from which the five best IDs were extracted (Table S5 in the Supplementary Materials) and re-optimised using QE. Of these, only two IDs (Table 4) met the selection criteria described above. From ^13^C and ^1^H RMSEs and energy evaluation it is clear that the most encouraging structure is ID 80, displaying a better RMSE value as well as a lower energy.

**Figure 12:**
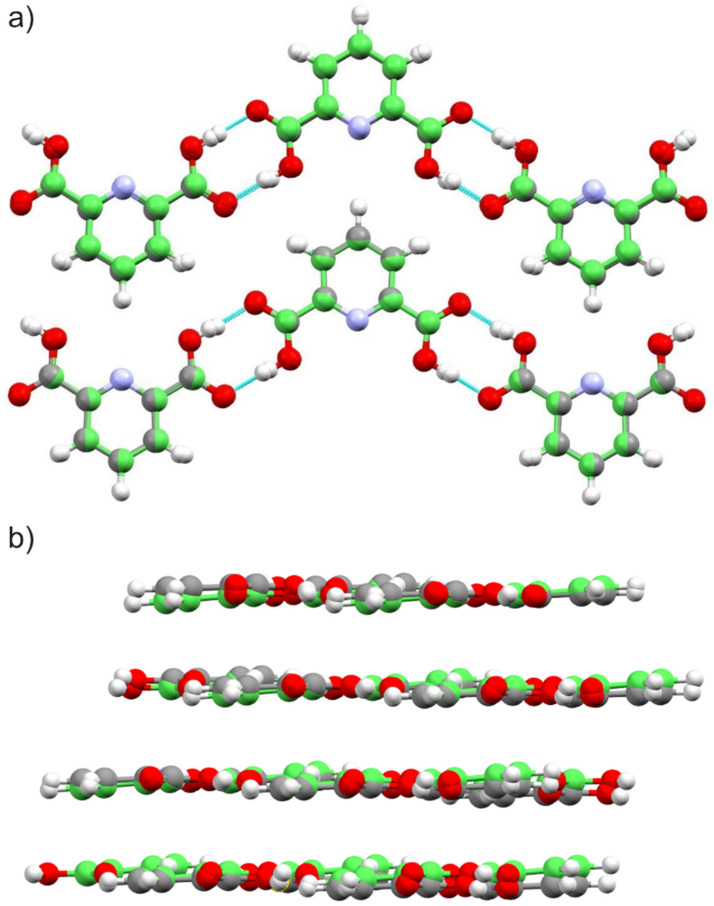
Overlay of the experimental crystal structure of DPA (standard colours: C = grey, O = red, N = blue, H = white, hydrogen bonds = turquoise) and the best predicted individual (ID 80, in green): (**a**) hydrogen-bond pattern and (**b**) molecular layers.

ID 80 was compared with the experimental structure of DPA, showing a perfect agreement in terms of spatial molecular arrangement and hydrogen-bond pattern. The overlay of the experimental and the predicted structures is represented in Figure 12, where the hydrogen-bond pattern and the molecular layers are shown. The cell parameters of the experimental and predicted crystal structure are shown in Table 5.

#### 2.2.3. CSP of DNic

Two preliminary CSP calculations were carried out employing USPEX with the Dreiding FF, using both the neutral and the zwitterionic DNic molecule as molecular input. In the first case, no plausible crystal structure was found. In the latter case, we found quite a good structure within the best predicted individuals, with space group *P*2_1_/*c* (*Z*′ = 1), reasonable cell parameters and hydrogen-bond network. The ^1^H, ^13^C, and ^15^N RMSE values of the computed and experimental SSNMR chemical shifts were 1.9, 1.7, and 47.5 ppm, respectively. The comparison with the experimental crystal structure is almost perfect but for a mismatch of the formed hydrogen-bonded ring (see Figure S8 in the Supplementary Materials). For this reason, and because of the uncertainty of the proton position, we performed a third CSP calculation but employing a DFT method for the geometry optimisation rather than the molecular mechanics (MM) method. USPEX produced 1108 structures that were grouped on the base of their crystal packing similarity. Of these, only two were selected as best IDs. The relative energies and ^13^C and ^1^H chemical shifts RMSEs with experimental values are reported in Table 6. Since SSNMR calculations are performed at 0 K, computed ^13^C chemical shifts were also compared with the experimental ones obtained at 173.15 K; as expected, in this case, we observed lower ^13^C RMSE values, as shown in Table 6.

From relative energies and chemical shifts RMSEs it is not possible to unambiguously select the correct crystal structure of DNic.

**Figure 13:**
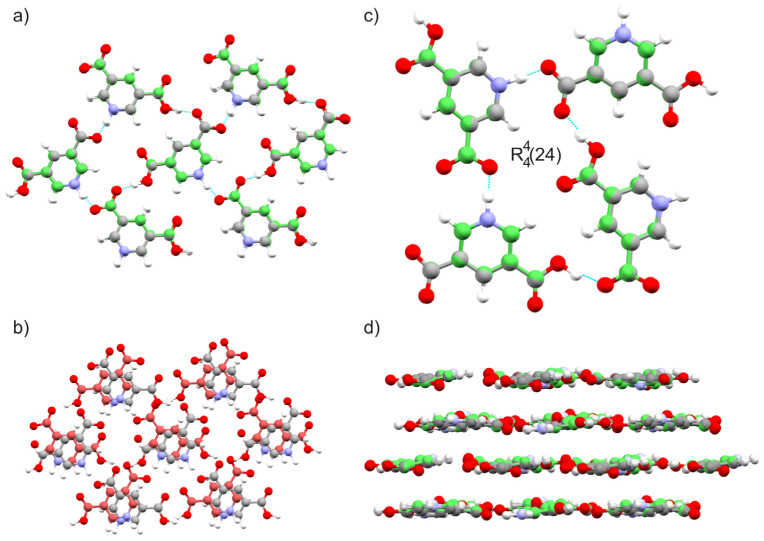
(**a**) Overlay of the molecular layer A of the predicted crystal structures of DNic, ID 715 (standard colours: C = grey, O = red, N = blue, H = white, hydrogen bonds = turquoise) and ID 791 (in green). (**b**) overlay of the molecular layer B of the predicted crystal structures of DNic, ID 715 (standard colours) and ID 791 (in red); overlay of the experimental crystal structure of DNic (15 K, standard colours) and the best predicted individual (ID 715, in green): (**c**) hydrogen-bond pattern and (**d**) molecular layers.

As can be observed in Figure 13a, both structures show molecular arrangements and the formation of an $R_{4}^{4}\left(24\right)$ hydrogen-bonded ring consistent with the information obtained from 2D SSNMR spectra (Figure 8), in particular from the H7/C4-C5 and H7/C8 correlations. The crystal structures of both ID 715 and ID 791 are characterised by planar sheets, which differ for the relative orientation of the molecules in two neighbouring layers, indicated as A and B in Figure 13. While layer A is the same in both IDs, layer B differs in the molecular orientation (Figure 13b).

**Figure 14:**
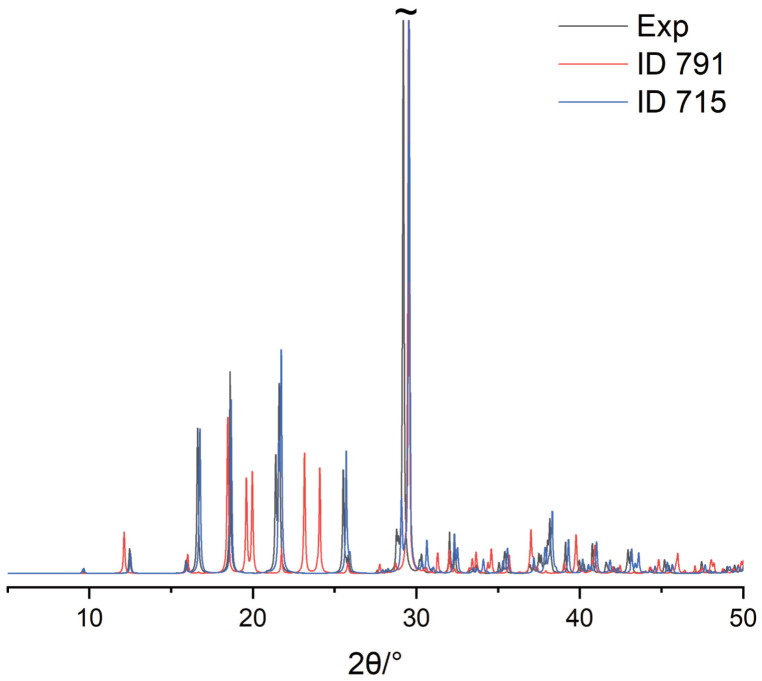
Overlay of the simulated powder patterns of the experimental crystal structure of DNic (PXRD, in black) with ID 715 (blue) and ID 791 (red).

Since ID 715 and ID 791 are very similar in terms of energy and chemical shift RMSEs, we relied on the ability of the ^1^H DQ MAS experiment in probing ^1^H-^1^H proximities, which strongly reflect the crystal packing, for selecting the best candidate. The presence of a weak correlation between H1 and H1 of two neighbouring molecules in the ^1^H DQ MAS spectrum of DNic (Figure S6 in the Supplementary Materials) seemed to point to ID 715 as the best one since it is characterised by the smallest distance (3.23 Å) between these two atoms with respect to ID 791 (3.77 Å). The selection was confirmed by the comparison among the simulated XRD powder patterns of the experimental (15 K) and the predicted crystal structures (Figure 14). The powder pattern of ID 715 is significantly much more consistent with the experimental one; the small difference in the peak positions is probably due to tiny variations of the cell parameters of the experimental and computed structures, while peak intensity is certainly comparable. Thus, ID 715 was selected as the correct crystal structure. Table 7 reports the cell parameters of the experimental and predicted crystal structures of DNic, which are in perfect agreement, as well as the overlay of the two structures (Figure 13c,d), which displays an RMSE of only 0.063.

In this case, the use of chemical shifts RMSEs was not sufficient to determine which of the two predicted structures was the correct one since they differed only for the orientation of the molecules in the alternating parallel layers. Indeed, ^13^C and ^1^H RMSEs are less sensitive to this kind of structural variation; thus, in this case, the comparison with experimental SSNMR data proves marginal in discriminating between the two predicted structures.

## 3. Materials and Methods

### 3.1. Materials

QA (99% purity), DNic (98% purity) and DPA (98% purity) were purchased from Alfa Aesar and used without further purifications.

### 3.2. SSNMR Experiments

All SSNMR spectra, except for ^1^H MAS spectra and the low-temperature ^13^C CPMAS spectrum of DNic, were recorded at room temperature on a Bruker Avance II 400 Ultra Shield spectrometer, operating at 400.23, 100.63 and 40.56 MHz for ^1^H, ^13^C and ^15^N, respectively. ^13^C and ^15^N CPMAS spectra were acquired with 4 mm zirconia rotors at a spinning speed of 12 (^13^C) and 9 (^15^N) kHz, using a ramp cross-polarisation pulse sequence (90° ^1^H pulse of 3.8 μs; contact time of 3 and 4–7 ms for ^13^C and ^15^N, respectively). ^1^H-^13^C 2D CP (“short range”) and LG-CP (“long range”) FSLG HETCOR measurements were acquired at a spinning speed of 12 kHz, with a contact time of 0.1 or 2.5 ms, to allow only for short-range or also long-range magnetisation transfer, respectively. For every spectrum, a two-pulse phase modulation (TPPM) decoupling scheme was used, with a radiofrequency field of 69.4 kHz.

^1^H MAS spectra and the low-temperature ^13^C CPMAS spectrum of DNic were acquired on a Jeol ECZR 600, operating at 600.17 MHz for ^1^H and 150.91 MHz for ^13^C. The ^1^H MAS spectra for DNic were acquired at room temperature with 1 mm zirconia rotors at a spinning speed of 62.5 kHz with an echo pulse sequence (90°-τ–180°-τ) to remove the probe background (^1^H 90° pulse = 0.9 μs, τ = 27 μs). The ^13^C CPMAS spectrum of DNic was acquired with a 3.2 mm zirconia rotor at 173.15 K (temperature calibrated on the ^207^Pb signal of Pb(NO_3_)_2_) [46] at a spinning speed of 20 kHz, using a ramp cross-polarisation pulse sequence with a 90° ^1^H pulse of 1.9 μs, a contact time of 3.5 ms. For the ^13^C CPMAS spectrum, a two-pulse phase modulation (TPPM) decoupling scheme was used, with a radiofrequency field of 108.5 kHz.

The 2D ^1^H DQ MAS experiment was performed with the back-to-back (BABA) recoupling pulse sequence with excitation time durations of one rotor period (^1^H 90° = 0.9 μs).

All recycle delays used in this work are optimised by means of saturation recovery experiments. Complete experimental parameters are reported in the Supplementary Materials (Table S1).

For the PM-S-RESPDOR experiment on DNic, we used the pulse sequence reported elsewhere [40]. The ^1^H radio frequency (rf) field for π/2 and π pulses was 278 kHz, while it was 125 kHz for the $\mathrm{SR}4_{1}^{2}$ recoupling sequence. The length of the PM pulse was 10*t*_R_ (0.16 ms) and the ^14^N rf field was 80 kHz (calibrated through NH_4_Cl). To reach the steady state, prior to the PM-S-RESPDOR measurements, nine dummy scans were applied. The mixing time (τ) was varied from 0 to 1.3 ms. The number of scans was 27, with a recycling delay of 200 s. The total experimental time was 90 h. Details of the procedure for the extraction of the N–H distance are reported in the Supplementary Materials. The ^1^H, ^13^C and ^15^N chemical shift scales were calibrated through the CH_2_ signal of external standard adamantane (at 1.87 ppm), the methylenic signal of external standard glycine (at 43.7 ppm) and the signal of external standard glycine (at 33.4 ppm with reference to NH_3_), respectively.

### 3.3. Crystal Structure Prediction

Molecular structures of QA, DNic, and DPA were optimised in gas phase using the program Gaussian 09 [47], employing the B3LYP functional with the optimised def2-SVP basis set [48,49], with no constraints imposed during geometry optimisations. The D3 version of Grimme’s dispersion method was applied adopting the Becke−Johnson damping scheme [50]. The nature of all stationary points was confirmed by normal-mode analysis. These optimised geometries were employed as initial molecules for the CSP with USPEX. The USPEX evolutionary algorithm [51,52] (v. 10.4) by Oganov’s group was used for the prediction of solid-state structures. The first generation of crystal structures was created using the random-sampling operator, while afterwards different variation operators were applied, in particular rot-mutation and lattice mutation. All the structures were sampled within a set of the most common observed space group for organic molecules, considering only one molecule in the asymmetric unit cell (*Z*′ = 1), as suggested from SSNMR spectra. For geometry optimisation steps of CSP of QA and DPA, GULP (v. 5.1) was applied, with the Dreiding [53] force field (FF). For DNic, due to the uncertainty of the protonation state (see text above), we used a DFT method for the geometry optimisation: VASP (v. 5.4.4) was employed with the included PAW-PBE pseudopotentials [54]. For each CSP calculation, only the structures resulting to be in the range of 10 kJ/mol from the lowest energy structure were taken into consideration. The best predicted structures were re-optimised at DFT level with Quantum Espresso (QE, v. 6.4.1) [55], employing the projector augmented wave (PAW) approach, with the non-local vdW-df2 method [56] and the B86r functional [57] with the SSSP set of pseudopotentials [58,59]. An energy cut-off of 60 Ry was used. For the best predicted structures, NMR calculations were performed using the Gauge Including Projected Augmented Wave (GIPAW) [60] and the PBE pseudopotentials from PS Library 1.0.0 [61] with an energy cut-off of 80 Ry, following the methodology previously described [62,63,64].

The theoretical absolute isotropic magnetic shielding (σ_iso_) values of the samples were converted into isotropic chemical shifts (δ_iso_) relative to the absolute magnetic shielding of the reference substance DABCO computed at the same level, applying Equation (1).

δ_iso_ (calc) = σ_iso_ (ref) − σ_iso_ (calc) + δ_iso_ (ref) (1)

δ_iso_ (ref) values of DABCO are 0.00, 47.74 and 10.97 ppm for ^1^H, ^13^C and ^15^N, respectively, while the absolute isotropic constant shieldings σ_iso_ (ref) are 27.03, 120.59 and 212.22 ppm for ^1^H, ^13^C and ^15^N, respectively, calculated from the reported neutron diffraction crystal structure [65].

### 3.4. Crystal Structures Comparison and Visualisation

For the visualisation and comparison of the predicted structures, the CCDC Mercury utility (v. 2020.2) was employed, using the crystal packing similarity method. The size of the molecular cluster was set to 15, while tolerance on angles and distances was set to 40%.

## 4. Conclusions

The aim of this work was to test the CSP-NMRX method on three of the structural isomers of pyridine dicarboxylic acid with ambiguous hydrogen positions, namely QA, DPA and DNic. Indeed, each of these molecules could be potentially zwitterionic or not in the solid state. Mono- and bidimensional SSNMR spectra (i.e., ^1^H MAS, ^13^C and ^15^N CPMAS, ^1^H DQ MAS, ^1^H-^13^C HETCOR and ^14^N-^1^H PM-S-RESPDOR) proved fundamental to determine the nature of QA, DPA and DNic in the solid state, providing exact information on the proton position, to be used as constraints in the input step of the CSP calculations. QA resulted to be zwitterionic and DPA non-zwitterionic. DNic was undoubtedly the most challenging system. SSNMR provided, through the ^14^N-^1^H PM-S-RESPDOR experiment, very precise indications that DNic is in a continuum at ambient conditions, while, with lowering the temperature, the migration of the proton from the carboxylic group to the pyridinic nitrogen becomes more pronounced. Furthermore, 2D SSNMR data unambiguously defined the correct isomer of QA in the unit cell, since the carboxylate could be in the ortho as well as in the meta position. All the information obtained from SSNMR experiments was used as input for the CSP calculations, allowing us to save a lot of computational time. CSP calculation provided several possible crystal structures which were selected according to the comparison of computed and experimental ^1^H and ^13^C chemical shifts (RMSEs). Regarding DNic, the SSNMR RMSE values were not sufficient to discriminate the correct crystal packing, due to their low sensitivity to small structural variations. In this case, the ^1^H DQ MAS experiment and the comparison of the calculated diffraction patterns of the predicted individuals with that of the experimental crystal structure were employed to pinpoint the right one. We strongly believe that the CSP-NMRX approach can be easily applied to a wide range of systems (from organic and organometallic to inorganic ones) which exhibit uncertain proton positions, and for which the crystal structure cannot be solved by diffraction methods, thus proving to be of great interest to the scientific community.

## Figures and Tables

**Table 1:** Comparison between the hydrogen bond interatomic distances between heavy atoms (Å) in the experimental crystal structures and the corresponding ^1^H chemical shift in QA, DPA and DNic.

	O∙∙∙O (Å)	δ ^1^H (ppm)	O∙∙∙N (Å)	δ ^1^H (ppm)
QA	2.399	20.4	2.725	14.7
DPA	2.625	14.2		
DNic	2.594 ^a^	12.8	2.515 ^a^	19.5

^a^ Values obtained from the experimental crystal structure acquired at room temperature.

**Table 2:** Energy differences (∆E, kJ/mol) and space groups of the best predicted structures of QA optimised with the Dreiding FF (top); energy differences (∆E, kJ/mol), ^13^C and ^1^H chemical shift RMSEs (ppm) for the best predicted structures of QA optimised at DFT level with QE (bottom).

ID	ΔE Dreiding FF (kJ/mol)	Space Group	
315	0.0	*P*2_1_ 2_1_ 2_1_ (19)	
1059	7.3	*P*$\bar{1}\;$(2)	
630	8.0	*P*2_1_/*c* (14)	
567	8.3	*P*$\bar{1}\;$(2)	
**ID**	**ΔE PBE vdW-df2** **(kJ/mol)**	**^13^** **C RMSE** **(ppm)**	**^1^** **H RMSE** **(ppm)**
315	0.00	2.8	0.6
630	4.66	1.8	0.4

**Table 3:** Unit cell parameters of the experimental (Exp) and predicted crystal structures (ID 630 and ID 315) of QA.

	Space Group	Volume/Å^3^	a/Å	b/Å	c/Å	α/°	β/°	γ/°
**Exp**	*P*2_1_/*c* (14)	658.495	7.4218	12.7050	7.8342	90.000	116.950	90.000
**ID 630**	*P*2_1_/*c* (14)	653.542	7.8209	12.5746	8.0365	90.000	124.218	90.000
**ID 315**	*P*2_1_2_1_2_1_ (19)	663.373	14.0748	6.7988	6.9324	90.000	90.000	90.000

**Table 4:** Energy differences (∆E, kJ/mol) and ^13^C and ^1^H chemical shift RMSEs (ppm) for the best predicted structures of DPA, optimised both with Dreiding FF and at DFT level with QE.

ID	ΔE Dreiding FF (kJ/mol)	ΔE PBE vdW-df2 (kJ/mol)	Space Group	^13^C RMSE (ppm)	^1^H RMSE (ppm)
80	0.9	0.00	*P*2_1_/m (11)	1.1	1.4
448	0.0	7.91	*P*$\bar{1}\;$(2)	2.7	2.9

**Table 5:** Unit cell parameters of the experimental (Exp) and predicted crystal structures (ID 80 and ID 448) of DPA.

	Space Group	Volume/Å^3^	a/Å	b/Å	c/Å	α/°	β/°	γ/°
**Exp**	*P*2_1_/*m* (11)	338.666	3.7663	16.1667	5.5705	90.000	93.160	90.000
**80**	*P*2_1_/*m* (11)	324.968	3.6202	16.0829	5.5819	90.000	90.759	90.000
**448**	*P*$\bar{1}$ (2)	323.144	7.6977	7.4146	6.6543	70.364	83.685	110.698

**Table 6:** Energy differences (∆E, kJ/mol) and ^13^C and ^1^H chemical shift RMSEs (ppm) for the best predicted structures of DNic. The ^13^C RMSEs were computed using both room- and low-temperature experimental chemical shifts.

ID	ΔE PBE vdW-df2 (kJ/mol)	Space Group	^13^C RMSE 298 K (ppm)	^13^C RMSE 173.15 K (ppm)	^1^H RMSE (ppm)
715	0	*P*2_1_/c (14)	2.2	1.8	1.7
791	1.0	*P*2_1_/c (14)	1.5	1.4	1.4

**Table 7:** Unit cell parameters of the experimental (Exp, 15 K) and predicted crystal structures of DNic.

	Space Group	Volume/Å^3^	a/Å	b/Å	c/Å	α/°	β/°	γ/°
**Exp**	*P*2_1_/*c* (14)	660.255	9.7116	11.1347	6.4421	90.000	108.600	90.000
**715**	*P*2_1_/*c* (14)	649.278	9.7225	11.1105	6.3827	90.000	109.583	90.000
**791**	*P*2_1_/*c* (14)	651.722	11.8823	11.0653	6.0679	90.000	125.226	90.000

## Data Availability

The data presented in this study are available in the main text and Supplementary Materials.

## Supplementary Materials

The following supporting information can be downloaded at: https://www.mdpi.com/article/10.3390/molecules28041876/s1, Figure S1: ^1^H MAS spectra; Figure S2: ^13^C CPMAS spectra; Figure S3: ^15^N CPMAS spectra; Figure S4: ^1^H-^13^C short-range HETCOR of QA; Figure S5: ^1^H-^13^C short-range HETCOR of DPA; Figure S6: ^1^H DQ MAS of DNic; Figure S7: ^1^H-^13^C short-range HETCOR of DNic; Figure S8: overlay of experimental and predicted (Dreiding FF) crystal structures of DNic; Table S1: complete SSNMR experimental parameters; Table S2: experimental and computed chemical shifts of QA; Table S3: experimental and computed chemical shifts of DPA; Table S4: experimental and computed chemical shifts of DNic; Table S5: relative energies of DPA predicted structures with the Dreiding FF.
