# Supplementary material for: Zwitterionic or Not? Fast and Reliable Structure Determination by Combining Crystal Structure Prediction and Solid-State NMR
Source: Molecules. 2023 Feb 16;28(4):1876. doi: 10.3390/molecules28041876 (PMC9966216; doi:10.3390/molecules28041876)
Supplement: Supplementary file 1 [file molecules-28-01876-s001.zip › molecules-2194486-supplementary.pdf]

ZWITTERIONIC OR NOT? FAST AND RELIABLE STRUCTURE DETERMINATION BY COMBINING CRYSTAL STRUCTURE PREDICTION AND SOLID-STATE NMR

Federica Bravetti, Raffaele Russo, Simone Bordignon, Angelo Gallo, Federica Rossi, Carlo Nervi, Roberto Gobetto and Michele R. Chierotti*

Experimental procedure for the extraction of the N···H distance from the ^{14}N-^{1}H PM-S-RESPDOR experiment

The ^{14}N-^{1}H PM-S-RESPDOR experiment is performed at high spinning frequencies (62.5 kHz) and is based on the detection of the ^{1}H signal of the proton in the ambiguous position (*i.e.,* H1 in DNic whose ^{1}H signal is at 19.6 ppm). Basically, the signal is acquired twice: in a first experiment, the intensity $S_0(\tau)$ of the ^{1}H signal is recorded with no irradiation on the ^{14}N channel; in a second experiment, the intensity $S'(\tau)$ derives from the employment of a ^{14}N PM-pulse preventing ^{1}H-^{14}N dipolar coupling from being refocused. Fraction curves can thus be obtained by plotting the quantity $(S_0(\tau) - S'(\tau))/S_0(\tau) = \Delta S/S_0$ against the mixing time, τ. A fitting of the curve is then performed (Figure 7 in the main text), which provides a value for the ^{1}H-^{14}N dipolar coupling constant ($b_{1H\text{-}14N}/(2\pi)$). Finally, the corresponding ^{1}H-^{14}N distance can be calculated by means of the following equation:

$$^{1}\text{H-}^{14}\text{N distance / Å} = \left(\frac{120.1}{b_{^1H-^{14}N}/(\frac{2\pi}{\text{kHz}})} \frac{\gamma_{^{14}N}}{\gamma_{^1H}} \right)^{1/3}$$

where γ_X ($X = {}^{1}H$ or ^{14}N) stands for the magnetogyric ratio of the X nucleus.

Table S1. ^{1}H MAS, ^{13}C and ^{15}N CPMAS, ^{1}H-^{13}C short- and long-range HETCOR and ^{1}H DQ MAS experimental parameters and line broadening used during processing for DPA, DNic and QA.

Experiment	Parameters	DPA	DNic	QA
**^{1}H MAS**	n. scans	3	3	3
	acquisition time (ms)	8.2	8.2	8.2
	relaxation delay (s)	161	40	300
	line broadening (Hz)	20	20	20
**^{13}C CPMAS**	contact time (ms)	3.5	3.5	3.5
	n. scans	100	184	36
	acquisition time (ms)	30	30	30
	relaxation delay (s)	161	40	300
	resolution (Hz)	33	33	33
	line broadening (Hz)	40	40	40
**^{15}N CPMAS**	contact time (ms)	7	3	4
	n. scans	700	2228	351
	acquisition time (ms)	35	35	35
	relaxation delay (s)	161	40	300
	resolution (Hz)	29	29	29
	line broadening (Hz)	30	30	30
**^{1}H - ^{13}C short range HETCOR**	contact time (ms)	0.1	0.1	0.1
	n. scans	450	300	680
	acquisition time (ms)	15	15	15
	relaxation delay (s)	10	10	10
	n. increments	19	42	42
	line broadening (Hz)	41	41	41
**^{1}H - ^{13}C long range HETCOR**	contact time (ms)	2.5	2.5	2.5
	n. scans	800	600	340
	acquisition time (ms)	12	12	12
	relaxation delay (s)	10	10	10
	n. increments	42	42	42
	line broadening (Hz)	60	60	60
**^{1}H DQ MAS**	n. scans	16	12	-
	acquisition time (ms)	8.2	8.2	
	relaxation delay (s)	190.5	200	-
	n. increments	48	32	-
	line broadening (Hz)	20	20	

Table S2. ^{1}H, ^{13}C and ^{15}N chemical shifts (ppm) of QA with assignments. For atom numbering please refer to Scheme 1 in the main text.

# atom	group	δ ^{13}C (ppm)		δ ^{1}H (ppm)		δ ^{15}N (ppm)	
		Exp	Calc	Exp	Calc	Exp	Calc
**1**	N(Ar)			14.7	15.0	205.3	209.8
**2**	C_q	143.5	141.0				
**3**	C_q	132.4	133.9				
**4**	CH	131.5	131.0	8.0	8.2		
**5**	CH	153.1	156.1	8.8	9.3		
**6**	CH	148.1	145.7	8.8	8.4		
**7**	COO^-	165.3	165.3				
**8**	COOH	160.4	161.1	20.4	19.9		
**RMSE**		1.8		0.4		4.5	

Table S3. ^{1}H, ^{13}C and ^{15}N chemical shifts (ppm) of DPA with assignments. For atom numbering please refer to Scheme 1 in the main text.

# atom	group	δ ^{13}C (ppm)		δ ^{1}H (ppm)		δ ^{15}N (ppm)	
		Exp	Calc	Exp	Calc	Exp	Calc
**1**	N(Ar)					316.0	325.7
**2**	C_q	145.9	144.6				
**3**	CH	130.8	130.3	7.4	8.7		
**4**	CH	141.2	140.4	7.4	8.2		
**5**	CH	130.3	130.2	7.4	8.7		
**6**	C_q	145.3	144.6				
**7**	COOH	171.2	172.9	14.2	12.5		
**8**	COOH	171.2	172.9	14.2	12.5		
**RMSE**		1.1		1.4		9.7	

Table S4. ^{1}H, ^{13}C and ^{15}N chemical shifts (ppm) of DNic with assignments. For atom numbering please refer to Scheme 1 in the main text.

# atom	group	δ ^{13}C (ppm)			δ ^{1}H (ppm)		δ ^{15}N (ppm)	
		Exp 298 K	Exp 173.15 K	Calc	Exp	Calc	Exp	Calc
**1**	N(Ar)				19.5	18.2	252.9	223.5
**2**	CH	150.8	150.4	148.6	8.6	7.8		
**3**	C_q	131.7	132.3	133.5				
**4**	CH	142.2	142.6	141.6	8.6	8.0		
**5**	C_q	130.5	131.1	132.1				
**6**	CH	147.2	146.4	143.2	8.6	7.9		
**7**	COOH	162.9	163.1	165.1	12.8	16.1		
**8**	COO^{-}	169.0	169.3	170.1				
**RMSE**		2.2 (Exp 298 K)			1.7		29.4*	

*The high ^{15}N RMSE value is expected, as the computed chemical shift is due to a protonated nitrogen (DNic is zwitterionic at very low temperature and calculation is performed at 0 K), while the experimental one refers to a pyridinic nitrogen involved in a very strong hydrogen bond, with the proton shared between the nitrogen and the carboxylic group. Indeed, DNic is in a continuum state at room temperature, at which the ^{15}N CPMAS spectra were acquired.

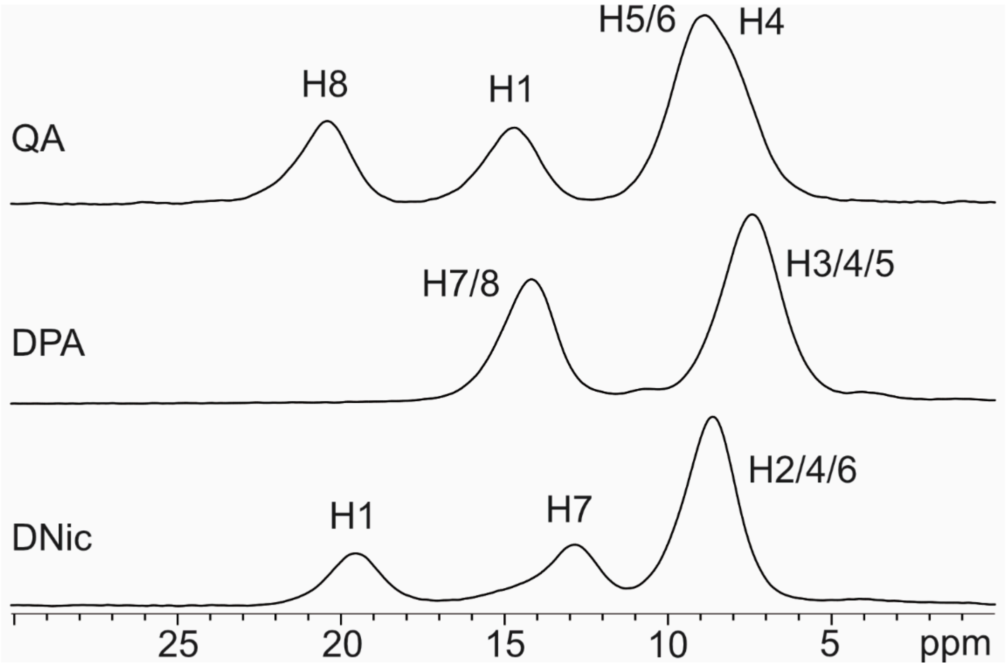

Figure S1. ^{1}H (600.17 MHz) MAS spectra with assignments of QA, DPA and DNic, acquired at room temperature at a spinning speed of 50, 68 and 60 kHz, respectively. Atom numbering refers to Scheme 1 in the main text.

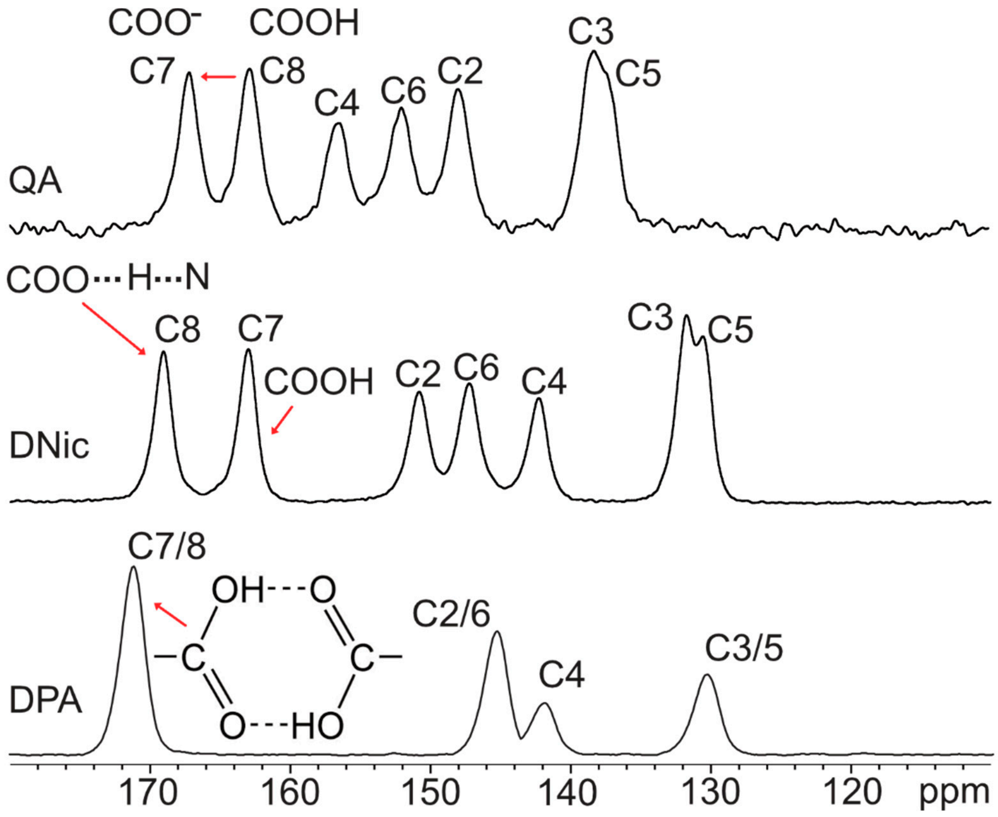

Figure S2. ^{13}C (100.63 MHz) CPMAS spectra with assignments of QA, DNic and DPA acquired at room temperature at a spinning speed of 12 kHz. Atom numbering refers to Scheme 1 in the main text.

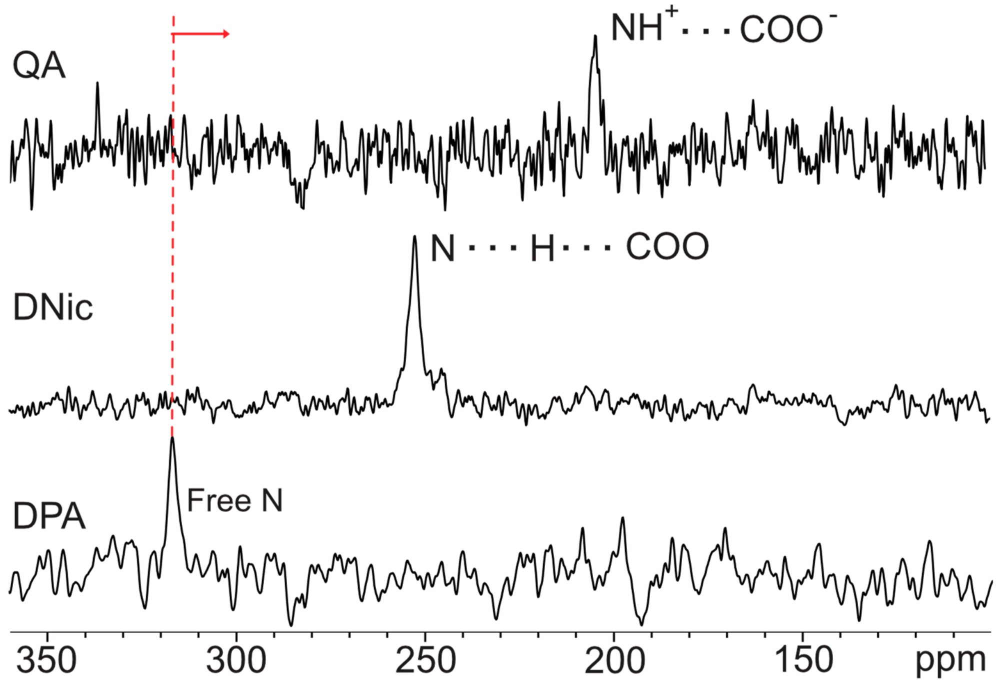

Figure S3. ^{15}N (40.56 MHz) CPMAS spectra with assignments of QA, DNic and DPA acquired at room temperature at a spinning speed of 9 kHz. Atom numbering refers to Scheme 1 in the main text.

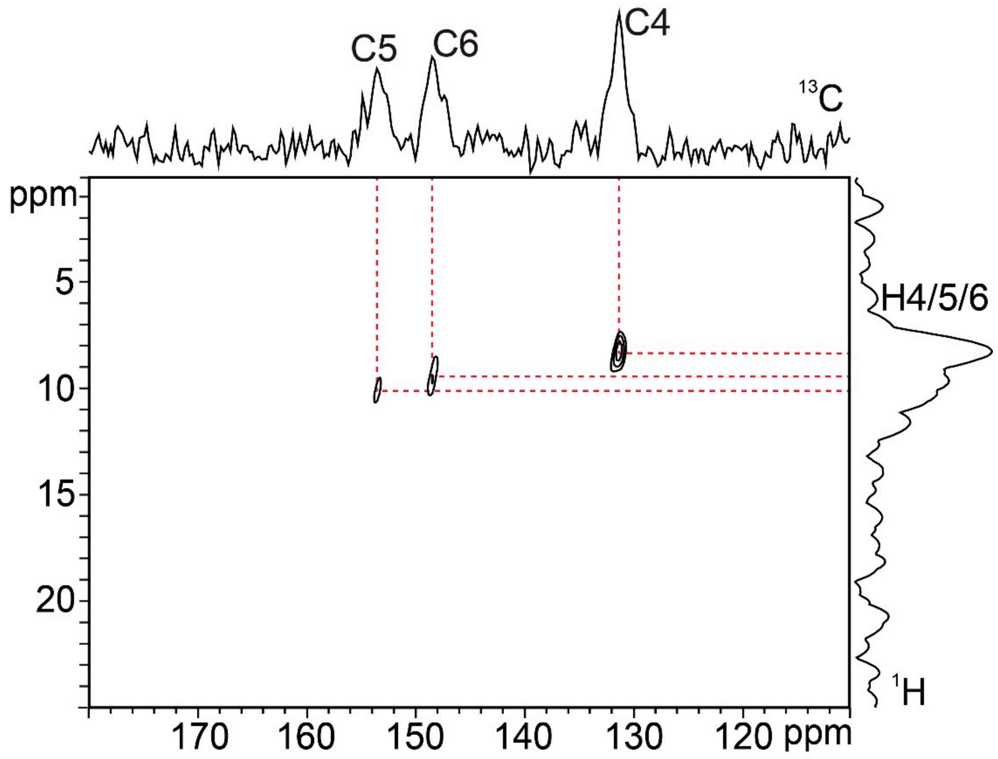

Figure S4. ^1H-^{13}C short-range HETCOR of QA with assignments. For atom numbering please refer to Scheme 1 in the main text.

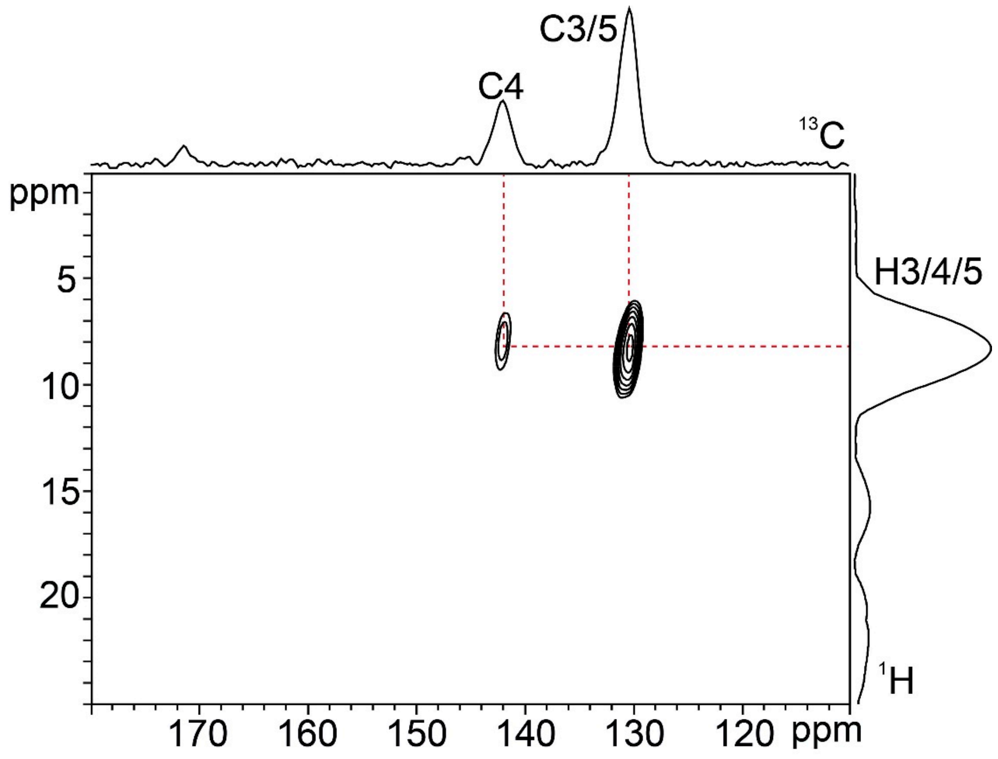

Figure S5. ^1H-^{13}C short-range HETCOR of DPA with assignments. For atom numbering please refer to Scheme 1 in the main text.

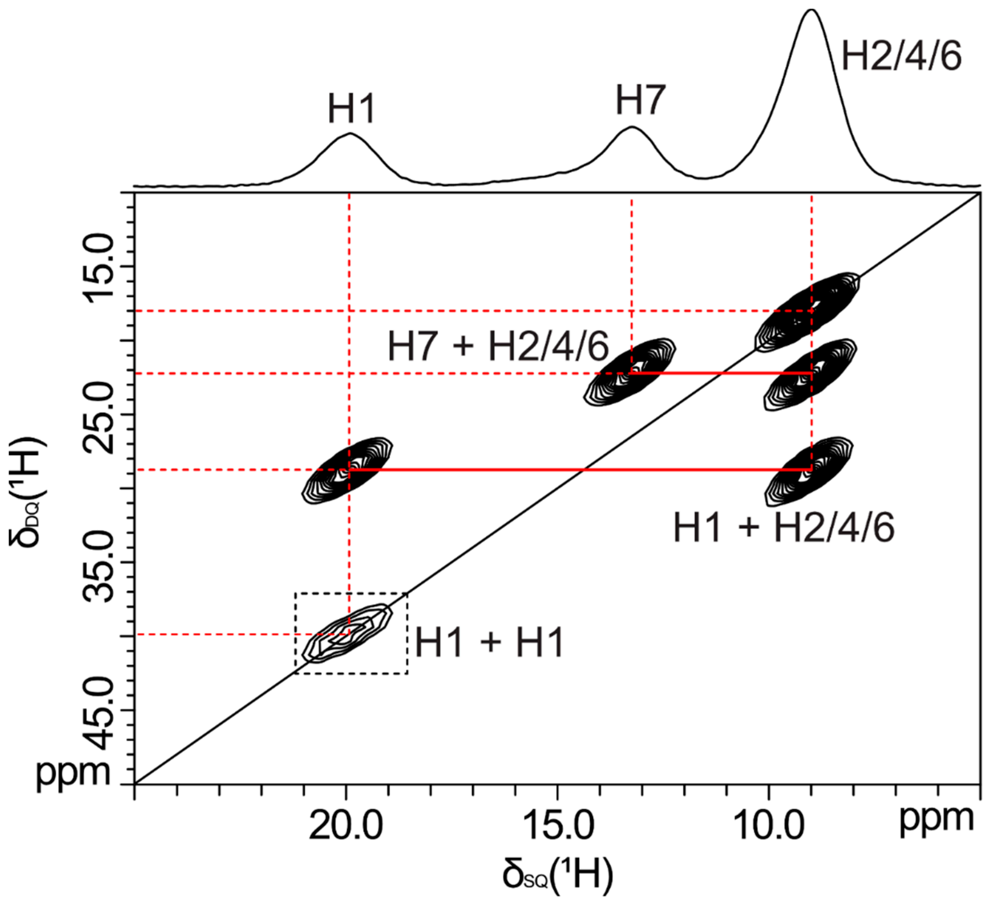

Figure S6. ^{1}H (600.17 MHz) DQ MAS SSNMR spectrum of DNic recorded at 60 kHz. The dashed box (magnification of 4x) highlights the correlation between H1 and H1 of two neighbouring molecules which helped to select ID 715 as the best candidate.

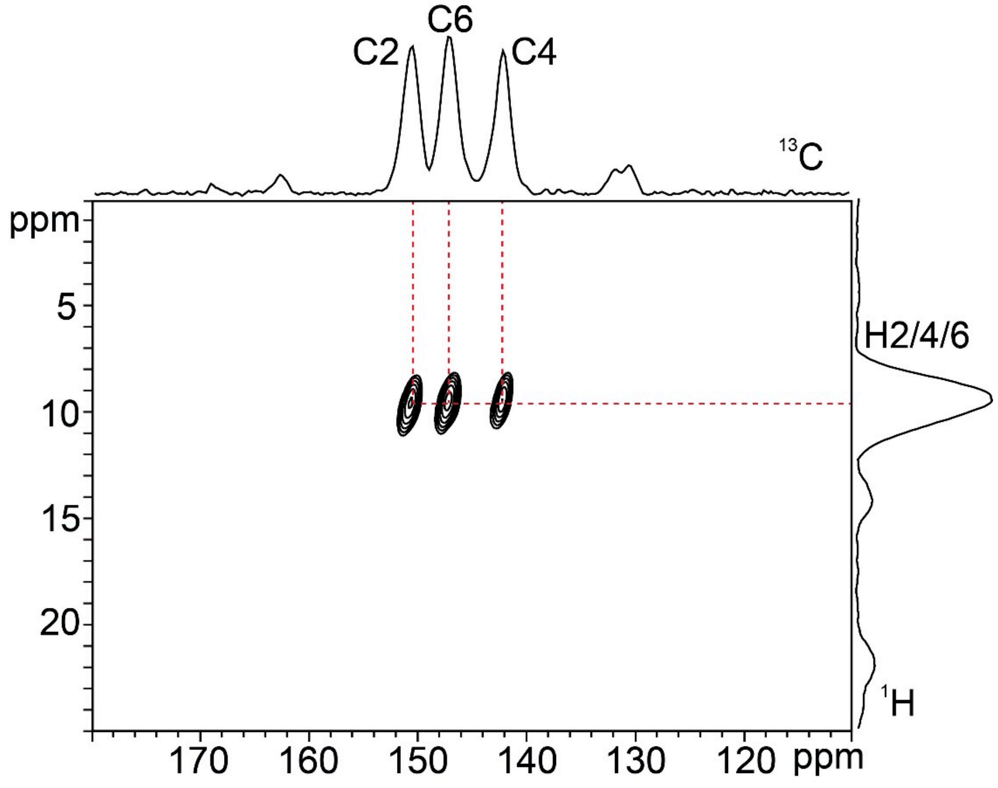

Figure S7. ^{1}H-^{13}C short-range HETCOR of DNic with assignments. For atom numbering please refer to Scheme 1 in the main text.

Table S5. Relative energies (kJ/mol) and space groups of the best predicted structure for DPA, optimised with the Dreiding force field.

Individual	ΔE Dreiding FF (kJ/mol)	Space Group
**448**	0.0	P $\bar{1}$ (2)
**80**	0.9	P 2_1/m (11)
**208**	5.5	P c (7)
**175**	7.9	P 1 (1)
**806**	8.1	P 2_1 (4)

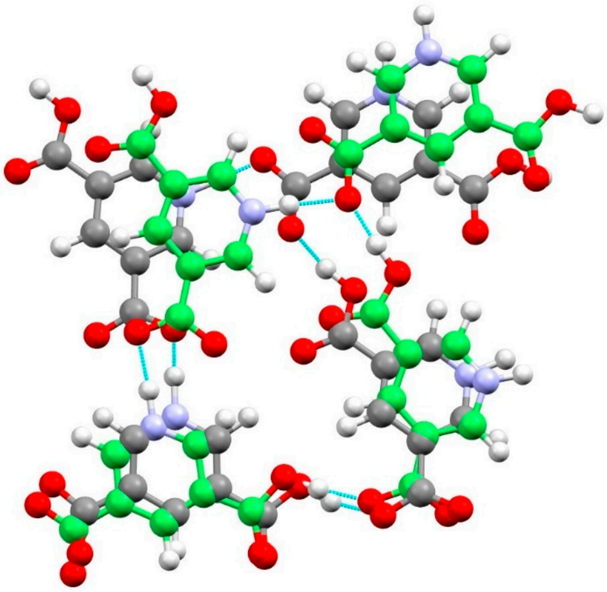

Figure S8. Overlay of the hydrogen-bonded ring formed in the experimental (15 K, in standard colours) and predicted (USPEX + Dreiding, carbon atoms in green) crystal structure of DNic.
